# Supplementary material for: Cell Profiling of Acute Kidney Injury to Chronic Kidney Disease Reveals Novel Oxidative Stress Characteristics in the Failed Repair of Proximal Tubule Cells
Source: Int J Mol Sci. 2023 Jul 18;24(14):11617. doi: 10.3390/ijms241411617 (PMC10380716; doi:10.3390/ijms241411617)
Supplement: Supplementary file 1 [file ijms-24-11617-s001.zip › ijms-2393050-supplementary-figure.pdf]

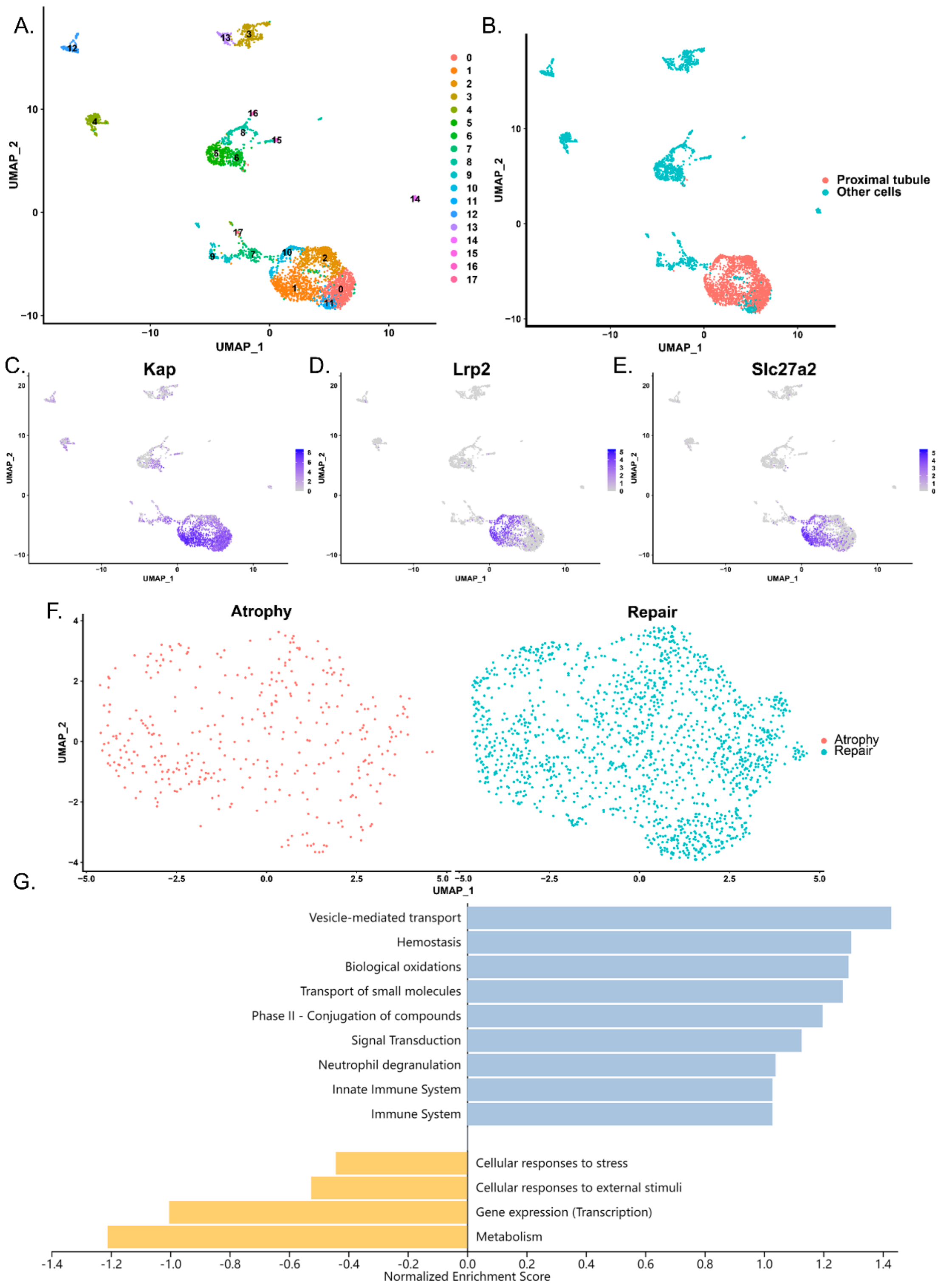

Supplementary Figure S1. Single-cell RNA sequencing (scRNA-seq) identifies the characterization of PT cells in repaired and atrophied kidneys. (A) unsupervised clustering identified 17 distinct clusters in the UMAP plot. (B) Renal proximal tubular epithelial cells were identified

with red plots in the UMAP. (C)-(E) UMAP plots show the expression of the proven marker genes in PT cells, including (C) Kidney androgen-regulated protein (Kap), (D) LDL Receptor Related Protein 2 (Lrp2), (E) Solute Carrier Family 27 Member 2 (Slc27a2). (F) Renal proximal tubular epithelial cells in atrophied and repaired kidney samples were respectively identified with red (atrophied sample) and blue (repaired sample) plots in the UMAP. (G) The Gene Set Enrichment Analysis (GSEA) of the differential expressed genes between the atrophied sample and the repaired sample. The blue bars represent the up-regulated pathways in the atrophied sample, while the orange bars represent the up-regulated pathways in the repaired sample. All the pathways shown in this figure occupied a significant difference ($P<0.05$).

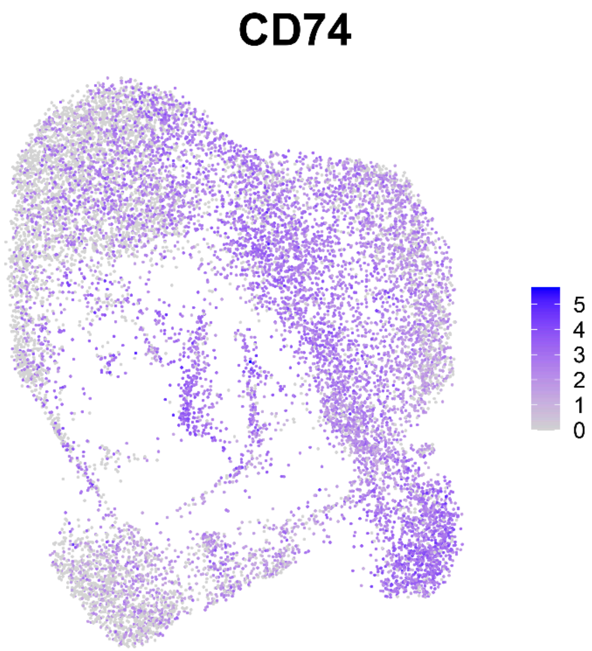

Supplementary Figure S2. CD74 was highly expressed where failed repair PT signature genes highly expressed in PT cells.

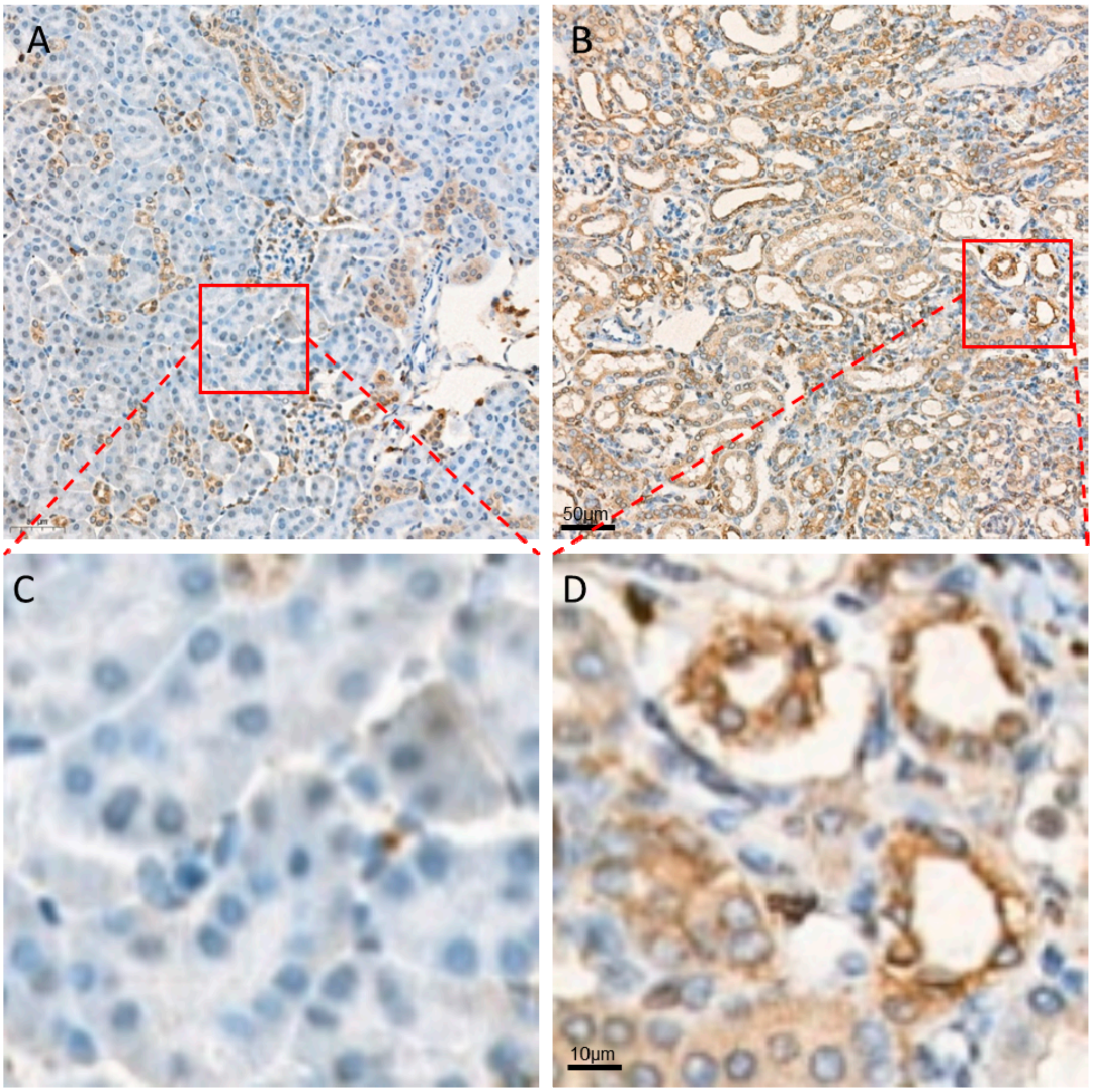

Supplementary Figure S3. IHC indicates that the CD74 expresses higher in the kidneys of UIRI mice. (A) The expression of CD74 in the kidneys of sham mice. The scanning magnification is 20×. (B) The expression of CD74 expresses in the kidneys of UIRI mice. (C) Higher magnification view of (A). (D) Higher magnification view of (B).

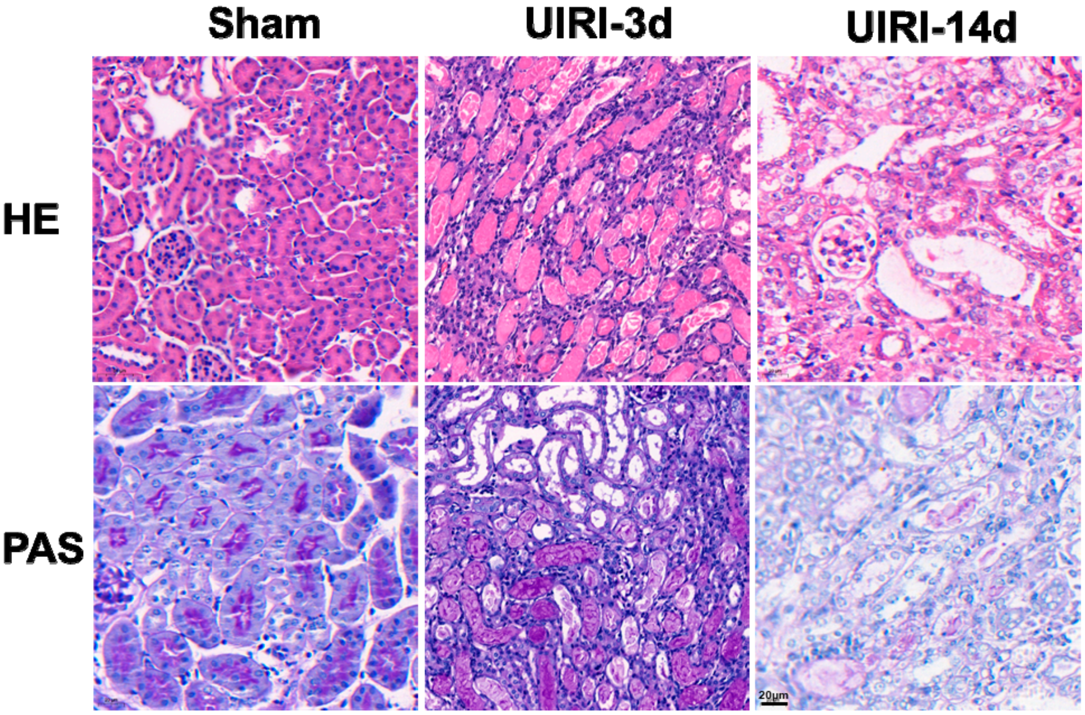

Supplementary Figures S4 Tissue analysis (HE and PAS) for the UIRI model.

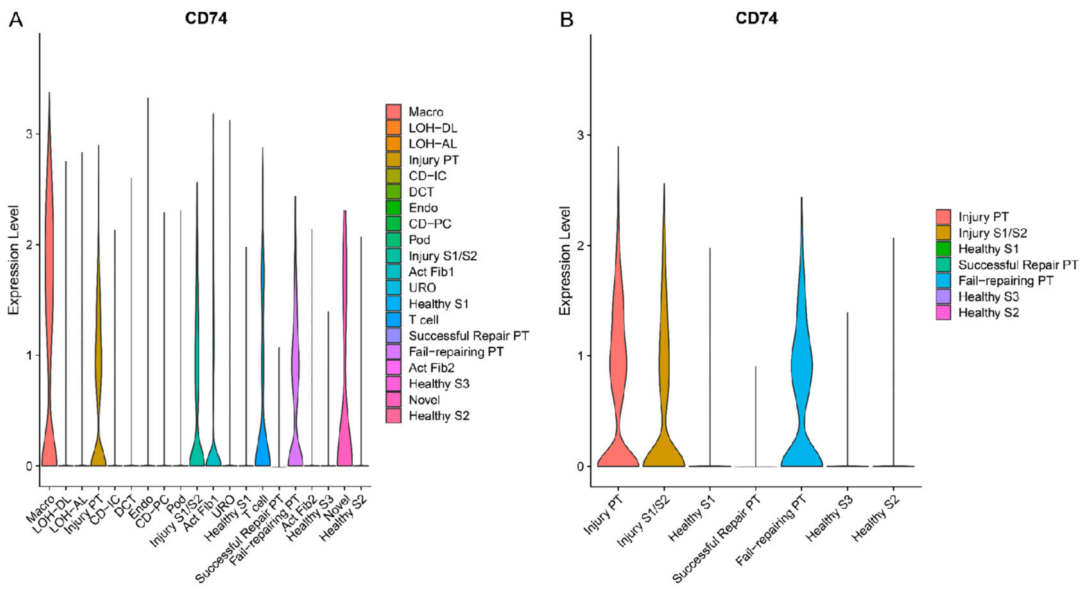

Supplementary Figures S5 The expression level of CD74 in (A) all kinds of cells and (B) in different PT cell subtypes.
