# Supplementary material for: Cell Profiling of Acute Kidney Injury to Chronic Kidney Disease Reveals Novel Oxidative Stress Characteristics in the Failed Repair of Proximal Tubule Cells
Source: Int J Mol Sci. 2023 Jul 18;24(14):11617. doi: 10.3390/ijms241411617 (PMC10380716; doi:10.3390/ijms241411617)
Supplement: Supplementary file 1 [file ijms-24-11617-s001.zip › ijms-2393050-supplementary-tables.pdf]

Genes in different clusters in BEAM analysis	
Gene	Cluster
Sdc4	1
Adgrg2	2
Selenop	1
Ifrd1	2
Ccdc141	1
Tgfbr3	2
Dbi	2
Hivep1	2
Itgb6	2
Mpc2	1
Gtdc1	2
Galnt18	1
Nfkbiz	2
Ppm1l	1
Prickle2	2
Tnrc18	1
Hnf1b	1
Col4a2	2
BC005537	3
Limch1	1
Adgrl2	2
Kcnh8	2
P4ha1	1
Tln2	1
Parp14	2
Atp6v1e1	1
Pdlim5	2
Ypel2	2
Ankrd12	2
Arhgap6	1
Zbtb16	2
Chka	2
Large1	2
Gpc3	2
Sash1	2
Etv6	2
Tcf4	2
Ivns1abp	1
Dstn	2
Adamts9	2
Pdia3	3

Trpm7	1
Kcnc2	2
Hook3	3
Osbpl3	2
Tnfaip3	2
Peli2	1
Rims2	2
Ppp4r4	2
Stxbp6	2
Neat1	2
Ipmk	1
Gsap	2
Adam10	2
Nckap5	2
Clic4	2
Atp13a3	2
Erbin	3
Pfkfb3	2
Abtb2	2
Ctsl	2
Eda	2
Phldb2	1
Ccdc148	2
Sh3d19	2
Sgms2	2
Zfp704	2
Dach1	2
Tle4	1
Aff1	3
Oxct1	1
Ankrd44	2
Smad7	3
Shroom4	1
B4galt5	2
Myo5b	2
Atp5a1	3
Fras1	1
Kansl1	1
Hnf1aos1	2
Rnf180	2
Ube2e2	2
Slc30a5	1
Shank2	1

Pclo	1
Pax8	2
Slc6a18	1
Arhgap26	2
2210408I21Rik	1
Prdx1	3
Mid1	2
Pkp4	1
Rnf128	3
Airn	2
Commd10	1
2610307P16Rik	2
Cgnl1	3
Cobl	2
Fam107b	1
Me3	1
Gcc2	3
Cox7c	3
Iqgap2	1
Gcnt2	2
Wdr72	1
Arid5b	2
Rdh10	2
Mpp6	2
Zfp36l1	2
Dock8	3
Gm4876	2
Abca1	2
Apbb2	1
Stat1	1
Fermt1	2
Pth1r	3
Ctsb	3
Prrg4	2
Ank3	1
Myo9a	2
Glis3	2
Cdk8	1
Wee1	2
Nrg1	2
Cobll1	1
Kif5b	3
Col4a1	2

Tbc1d1	2
Sptan1	1
Etnk1	3
Cd63	3
Pard3b	2
Resf1	2
Tmcc3	2
Itga6	2
Pde4b	2
Sema6a	2
Ldlrad4	2
Npnt	2
Ell2	1
Sema3c	2
Car2	1
Plau	2
Cdkal1	1
Ngf	2
Hivep2	2
Jun	2
Atp5b	3
Grip1	2
Mrpl33	2
C3	2
Trpm3	2
Setbp1	2
H3f3b	2
Map3k1	2
Veph1	2
Pik3c2a	2
Vmp1	2
Atp1b1	1
Slc12a2	3
5730522E02Rik	1
Gab2	3
Picalm	2
Rbpms	2
Prrg1	2
Trio	2
Nhsl2	2
Pbx1	1
Hs2st1	3
Cxcl2	2

Mdm2	3
Dapk1	3
Exoc4	3
Rbbp6	3
Agps	2
Eif2s2	3
Ext1	1
Psap	3
Tpr	3
Mast4	2
Slc25a30	1
Ptpn22	2
Camkmt	3
Enah	2
Birc3	2
Gpx3	1
Bod1l	3
Cfap69	1
Csgalnact1	1
Foxo3	1
Vgll4	1
Crim1	2
H2-K1	2
Rnf213	3
Lin52	1
Sdc2	1
Cltrn	1
Mllt3	3
Arhgap29	3
Znrf1	2
Cdh1	3
Itga1	2
Lpp	2
Ldhb	2
Shroom3	3
Slc22a23	1
Hspa5	3
Wsb1	3
Slco3a1	2
Birc2	2
Fhod3	2
Gclm	3
Atp1a1	1

Eif1	3
Cftr	2
Adamts1	2
Pabpc1	3
Dock4	1
St8sia6	2
Wwox	2
Acsl4	2
Plod2	2
Arhgap10	1
Pitpnc1	1
Pcdh7	1
Slc43a2	1
Rassf6	2
Nedd4l	1
Camk2d	3
Nfkb1	2
Hmgcs1	1
Meis2	3
Frk	2
Jarid2	1
Malt1	2
Vcl	2
Fat1	3
Cxcl1	2
Suz12	3
Zbtb38	3
Grk5	2
Gm15261	2
Ror1	2
Gls	3
Nudt4	3
Kcnma1	3
Fgf1	1
Hsp90b1	3
Ly6a	3
Plcl1	1
Mfhas1	2
Srrm1	3
Hmgb1	3
Kap	1
Gse1	3
Smarca2	1

Ly6e	2
Chn2	1
Txnip	3
9530026P05Rik	2
Ncl	3
Rpl5	3
Cmip	1
Zeb1	2
Nr3c2	3
Son	3
Itpr2	1
Slf1	3
Jazf1	2
Glul	1
Zfp697	1
Ppargc1a	1
Ybx1	3
Egfr	2
Fam129a	2
Rock2	3
mt-Nd3	3
Acyp2	1
Hspa8	3
Gxylt2	2
3-Mar	1
Tgfbr1	3
Phactr2	2
Chd4	3
Fth1	3
Lgmn	1
Tet2	1
Rpl17	3
Fam13b	2
Mme	3
Abcc4	3
Schip1	3
Pakap.1	2
Bcl2	3
Gm32461	2
Nrp1	3
Wwc1	3
Nfib	3
Cd9	2

Sema5a	2
Il34	2
Rpl27a	3
1600010M07Rik	2
Rras2	3
Fam13a	1
Uap1	2
Gmds	2
Cdh2	3
Ankrd33b	2
Prom1	2
Mapk4	2
Plscr1	2
Magi1	3
Fau	3
St3gal3	2
Dysf	3
Agbl4	1
Arl15	2
Pvt1	3
Slc7a8	1
Spns2	3
Syne2	3
Rbbp8	2
Ppia	3
Nt5e	2
Akt3	2
Tsc22d1	3
Paqr5	3
Chst11	1
Carmil1	3
Gm13415	2
Rpl23	3
Atp2a2	1
Prkag2	1
Arhgap24	1
Mylk	2
Smad3	3
Ptbp3	2
Phip	3
Eml4	3
Krt20	2
Tanc1	3

Rps12	3
Baz1b	3
Raph1	2
Palld	2
Ankrd6	2
Glud1	1
Vcam1	2
Srrm2	3
Cdk6	2
Rps2	3
Lgr4	1
Rpl10a	3
Plac8	2
Rbms1	3
Ctsd	3
Arsb	1
Npm1	3
Ugt8a	1
Zeb2	2
Parp8	2
Ppp2r3a	1
Sh3rf1	2
Cpe	2
Mcc	2
Ptprj	2
Cep112	3
Itgb1	2
Rpl13	3
Ranbp17	3
Rpl6	3
Gm13684	2
Gm42031	3
Tenm3	2
Fndc3b	3
Kl	1
Ntn1	2
Arhgap22	2
Dleu2	2
Sat1	2
Egfros	2
Pkhd1	2
Nt5c2	1
Esr1	2

Slc6a15	2
Kansl1l	2
Arhgap15	1
Msn	3
Asap1	2
Dnm3	3
Tfcp2l1	1
Dtnb	3
Rps20	3
Cadps2	2
Vav3	3
Mertk	1
Ptpn13	2
Ppp2r5e	1
Tbc1d4	3
Ccdc88a	3
Clstn2	3
Arhgef3	2
Hsp90ab1	3
Ccser1	3
Prkca	3
Itgav	2
Gclc	3
Hacl1	1
Rbm47	1
Nuak2	1
Cd2ap	2
Plch1	3
Slc6a6	1
1700025G04Rik	2
Met	3
Thsd7a	2
Tns3	2
Col4a4	1
Mir99ahg	1
Col18a1	2
Rps24	3
Pbx3	3
Bicc1	2
Mat2a	1
Gm16685	2
Ckap5	3
Chsy3	2

Slc16a10	1
Igfbp7	2
Erc2	1
mt-Nd4l	3
Rps8	3
Pank1	1
Rplp1	3
Nbea	3
Fhl1	1
Stard13	3
Gm12353	2
Macrod2	1
Actg1	3
Umod	3
Plcb4	3
Ftl1	3
Fxyd2	3
Kcnk5	1
Phyhipl	1
Peak1	3
Oxr1	3
Fkbp5	1
Scin	2
Wnk1	3
Hoxb6	3
Agmo	1
Tpt1	3
Sord	1
Hecw2	2
Lncpint	1
Pdgfd	2
Map3k7cl	1
Specc1	2
Gm20275	2
Cadm1	3
Rpl41	3
Dcdc2a	2
Hnf4g	1
Map3k5	1
Alcam	3
Tead1	3
Ptma	3
Actb	3

Cep85	2
Ezh2	3
1-Mar	2
Cyp4b1	1
Creb5	2
Atrx	3
Dst	3
Tmem178b	1
Gatm	1
Iqgap1	3
Kctd1	3
Gphn	1
Nfat5	2
Igsf11	1
Stk39	3
Gabrb3	1
Aoah	1
Plppr1	2
Gas2	1
Cttnbp2	2
Ncoa7	1
Anxa2	2
Nkain2	3
Maml3	3
Gm47283	3
Lrrfip1	3
Tnfaip8	1
Syn3	2
Pik3c2g	1
Map1b	2
Bcas3	1
Acot12	1
Eef1a1	3
Npas3	1
Phactr1	3
Slc16a12	1
Abcc2	1
Atxn1	1
Epb41	1
Rad51b	3
Tpm1	2
P3h2	1
Grid1	2

Sugct	1
Ptprm	3
Bmp6	2
Car12	1
Nsd2	3
Angpt1	1
Pawr	1
Samd4	2
Sorcs1	2
mt-Cytb	3
S100a6	3
Fmn1	3
Col4a3	1
Sycp3	2
Ppp1r16b	1
Pde1a	1
Plcb1	1
Gcnt1	1
Slc17a3	1
Etl4	2
Slc5a2	1
Gria3	1
Ptpn14	3
mt-Nd1	3
Rtn4	2
Anxa3	2
Cldn10	1
Immp2l	2
Sult1d1	1
mt-Nd5	3
Igf1r	1
Fam214a	1
Alpl	1
Slc1a1	1
mt-Nd2	3
Cpeb3	1
Tinag	1
Tns1	1
Slc2a2	2
Tacc2	1
Rora	2
Ncam1	3
Dock10	2

Cep128	3
Slc7a7	1
Nav2	3
Gm36388	2
Unc5c	1
Sytl2	2
Lpin2	1
Slc8a1	3
Adamts6	1
Slc12a3	3
Pdzrn3	1
Slit2	3
Gpc4	1
Pcsk5	2
Gm14764	1
Maml2	3
Lactb2	1
Clu	3
Srgap1	3
Ugt2b38	1
Myh9	2
Cpeb4	1
Thrb	1
Plat	2
mt-Nd4	3
Itgb8	2
Adra1a	1
Slc16a9	1
Aspa	1
Maf	1
Slc22a30	1
Plxdc2	1
Enpp2	1
Nfia	1
Hgd	1
Stox2	3
Cubn	1
Pah	1
Ahnak	3
Atp11a	2
Slc18a1	1
Sorbs2	3
Fyb2	2

Gm45792	1
Aass	1
Fmnl2	3
Osbpl6	1
Slc25a21	1
Naaladl2	3
Slc22a28	1
Tshz2	3
Pdzd2	1
Actn1	2
Pola1	3
Sdk1	3
Msra	1
Rgl1	1
Cald1	3
Lars2	3
Rbms3	3
Trps1	1
Myof	2
mt-Atp6	3
Glis1	1
Pdzk1	1
Cdh13	3
Cp	2
Hsd3b2	1
Prkn	1
Ar	1
Slc16a14	1
Cryl1	1
mt-Co3	3
Ntm	3
Timp3	2
Runx1	3
Epb41l3	1
Il7	2
Mki67	3
Trabd2b	1
Spp1	2
Frmd4b	1
Gpc6	3
Psd3	2
Fgfr2	2
Rhobtb1	2

Kcnj16	2
Aldob	1
Atp6v0a4	1
Slc12a1	3
Folh1	1
Gm42397	1
mt-Co2	3
Gramd1b	1
Tanc2	1
Slc17a1	1
Gm37245	2
Gldc	1
Wdr17	2
Utrn	3
Galnt14	1
Cd44	2
Erbb4	3
Gm20528	2
Esrrg	2
Camk1d	3
Pter	2
Lrp2	1
Them7	1
Slc5a12	1
Slc27a2	1
Snhg11	1
Hnf4aos	1
Pck1	1
Mecom	3
Dpyd	2
Fut9	1
Ptprd	1
Bnc2	1
Fggy	2
Cyp2j13	1
Cyp2j5	1
Errfi1	1
Magi2	2
Kcnip4	2
Ugt2b37	2
Slc4a4	1
Auts2	1
Slc13a1	2

Itpr1	1
Slco1a6	1
Nox4	1
Acsm2	1
Acsm1	1
Gm19950	1
Akap12	2
Chpt1	1
Nr1h4	1
Osbpl8	2
Havcr1	2
Taco1	3
Acox1	1
Slc34a1	1
Pde4d	1
Fhit	2
Lrmda	2
Gm6999	1
Diaph3	3
Ghr	1
Dab2	1
Gm16311	1

Supplementary Table S1 Genes in different clusters in BEAM analysis of SnRNA

DEGs in GSE161758							
	p_val	avg_log2FC	pct.1	pct.2	p_val_adj	cluster	gene
Tmsb4x	6.64E-25	1.613161	0.496	0.234	5.76E-21	atrophy	Tmsb4x
Gm11808	1.27E-24	1.149863	0.501	0.255	1.10E-20	atrophy	Gm11808
Apoe	3.49E-21	1.246883	0.345	0.137	3.03E-17	atrophy	Apoe
Cyba	2.05E-19	0.85564	0.633	0.394	1.78E-15	atrophy	Cyba
Igfbp7	1.01E-15	0.752096	0.592	0.385	8.73E-12	atrophy	Igfbp7
Ugt2b38	4.94E-15	1.095967	0.474	0.284	4.28E-11	atrophy	Ugt2b38
Lpl	2.06E-14	1.217384	0.285	0.126	1.79E-10	atrophy	Lpl
Defb29	6.22E-14	0.854035	0.51	0.329	5.39E-10	atrophy	Defb29
Cd36	1.19E-13	1.193577	0.255	0.108	1.03E-09	atrophy	Cd36
Tmem176a	2.48E-13	0.946866	0.458	0.29	2.15E-09	atrophy	Tmem176a
Tmem27	1.09E-12	0.732455	0.652	0.487	9.49E-09	atrophy	Tmem27
B2m	1.12E-12	1.009092	0.288	0.136	9.67E-09	atrophy	B2m
Cd63	6.98E-12	0.769585	0.529	0.35	6.05E-08	atrophy	Cd63
Tmbim4	1.32E-11	0.782129	0.405	0.242	1.14E-07	atrophy	Tmbim4
Cyp4b1	2.42E-11	0.907409	0.573	0.427	2.10E-07	atrophy	Cyp4b1
Ccdc107	3.32E-11	0.858919	0.37	0.211	2.87E-07	atrophy	Ccdc107
Arl6ip1	5.59E-11	0.767555	0.386	0.225	4.85E-07	atrophy	Arl6ip1
Lamp2	9.03E-11	0.723691	0.381	0.216	7.82E-07	atrophy	Lamp2
Bsg	3.44E-10	0.77416	0.323	0.177	2.98E-06	atrophy	Bsg

Slc27a2	4.01E-10	0.66804	0.567	0.426	3.48E-06	atrophy	Slc27a2
Ugt2b37	9.91E-10	1.008104	0.255	0.129	8.59E-06	atrophy	Ugt2b37
Kap	1.96E-09	0.812604	0.871	0.923	1.70E-05	atrophy	Kap
Cyp2j5	2.13E-09	0.608238	0.422	0.269	1.84E-05	atrophy	Cyp2j5
Actb	1.57E-08	0.649644	0.597	0.442	0.000136	atrophy	Actb
Cox6a1	3.37E-08	0.356539	0.811	0.755	0.000292	atrophy	Cox6a1
Napsa	5.13E-08	0.739863	0.373	0.242	0.000444	atrophy	Napsa
Tmem176b	5.44E-08	0.599625	0.466	0.332	0.000471	atrophy	Tmem176b
Spcs1	1.70E-07	0.673267	0.266	0.152	0.001472	atrophy	Spcs1
Lrpap1	2.48E-07	0.684109	0.263	0.15	0.00215	atrophy	Lrpap1
Pdzk1ip1	3.35E-07	0.660344	0.559	0.435	0.002902	atrophy	Pdzk1ip1
Atp1b1	5.24E-07	0.449329	0.559	0.425	0.004542	atrophy	Atp1b1
Nat8	7.33E-07	0.619163	0.326	0.209	0.00635	atrophy	Nat8
Acsm2	1.31E-06	0.490562	0.677	0.585	0.011348	atrophy	Acsm2
Psap	3.54E-06	0.496817	0.378	0.252	0.030674	atrophy	Psap
Sod3	4.76E-06	0.557646	0.266	0.162	0.041287	atrophy	Sod3
Cyp2e1	6.75E-06	0.707032	0.512	0.416	0.058533	atrophy	Cyp2e1
Ldhd	1.07E-05	0.501815	0.562	0.466	0.092847	atrophy	Ldhd
Timp3	1.60E-05	0.333641	0.471	0.333	0.13873	atrophy	Timp3
Ces1f	5.42E-05	0.491346	0.37	0.266	0.469706	atrophy	Ces1f
Folr1	6.26E-05	0.548554	0.367	0.261	0.542113	atrophy	Folr1
Guca2b	0.000121	0.327894	0.649	0.586	1	atrophy	Guca2b

Slc22a18	0.000188	0.426571	0.279	0.19	1	atrophy	Slc22a18
Atp1a1	0.000268	0.347985	0.452	0.345	1	atrophy	Atp1a1
Rps9	0.000302	0.312915	0.723	0.665	1	atrophy	Rps9
Smpdl3a	0.000592	0.561986	0.279	0.198	1	atrophy	Smpdl3a
Lamp1	0.001271	0.324236	0.405	0.332	1	atrophy	Lamp1
Ggt1	0.001528	0.453347	0.381	0.311	1	atrophy	Ggt1
Galnt11	0.001849	0.391672	0.337	0.256	1	atrophy	Galnt11
Hpn	0.002194	0.367734	0.258	0.183	1	atrophy	Hpn
Rps5	0.004116	0.283968	0.682	0.665	1	atrophy	Rps5
Hbb-bs	0.004913	0.585488	0.427	0.359	1	atrophy	Hbb-bs
G0s2	0.005778	0.586021	0.255	0.194	1	atrophy	G0s2
Pck1	2.48E-29	1.280063	0.765	0.507	2.15E-25	repair	Pck1
Sat1	1.75E-13	0.681086	0.652	0.452	1.52E-09	repair	Sat1
Bola3	4.42E-12	0.717431	0.542	0.351	3.83E-08	repair	Bola3
Iscu	9.17E-12	0.647751	0.606	0.427	7.95E-08	repair	Iscu
Pcbd1	1.14E-11	0.492953	0.71	0.507	9.89E-08	repair	Pcbd1
Cda	6.05E-11	0.486054	0.717	0.578	5.25E-07	repair	Cda
Cryl1	2.02E-10	0.625193	0.463	0.274	1.75E-06	repair	Cryl1
Sdhc	3.35E-10	0.623974	0.528	0.332	2.90E-06	repair	Sdhc
Ftl1	5.16E-10	0.351737	0.994	0.997	4.47E-06	repair	Ftl1
Ak4	6.20E-10	0.926664	0.31	0.145	5.37E-06	repair	Ak4
Idh1	8.84E-10	0.602014	0.556	0.373	7.66E-06	repair	Idh1

Bnip3	2.49E-09	0.769194	0.305	0.148	2.16E-05	repair	Bnip3
Fis1	2.61E-09	0.667793	0.436	0.255	2.26E-05	repair	Fis1
Scp2	2.87E-09	0.342703	0.887	0.841	2.49E-05	repair	Scp2
Cisd1	4.64E-09	0.48096	0.709	0.597	4.02E-05	repair	Cisd1
Gstm1	8.96E-09	0.765366	0.545	0.395	7.77E-05	repair	Gstm1
Akr1a1	9.22E-09	0.316896	0.944	0.901	7.99E-05	repair	Akr1a1
Pxmp4	1.26E-08	0.805993	0.283	0.14	0.000109	repair	Pxmp4
Gstt2	1.48E-08	0.598004	0.433	0.274	0.000128	repair	Gstt2
Atp5e	4.66E-08	0.29115	0.911	0.855	0.000404	repair	Atp5e
Dnajc19	7.34E-08	0.54587	0.536	0.373	0.000636	repair	Dnajc19
Gsr	8.97E-08	0.652136	0.36	0.211	0.000777	repair	Gsr
Fitm1	1.98E-07	0.701944	0.351	0.214	0.001718	repair	Fitm1
RP24-64C6-3	2.39E-07	0.524485	0.591	0.447	0.002067	repair	RP24-64C6-3
Atp6v1f	2.48E-07	0.502661	0.521	0.364	0.002152	repair	Atp6v1f
Prdx1	2.73E-07	0.34061	0.93	0.904	0.002368	repair	Prdx1
Uba52	2.94E-07	0.401713	0.769	0.693	0.002546	repair	Uba52
Med28	5.32E-07	0.567645	0.381	0.241	0.004613	repair	Med28
Serf2	7.38E-07	0.360822	0.804	0.699	0.006392	repair	Serf2
Hibadh	9.21E-07	0.469641	0.549	0.414	0.007985	repair	Hibadh
Lamtor2	1.12E-06	0.573747	0.4	0.258	0.009741	repair	Lamtor2
Mif	1.39E-06	0.373231	0.732	0.622	0.012066	repair	Mif
Slc25a5	1.79E-06	0.349314	0.747	0.677	0.015475	repair	Slc25a5

Atp5c1	1.98E-06	0.356074	0.64	0.507	0.017185	repair	Atp5c1
Sult1d1	4.36E-06	0.402399	0.639	0.545	0.037802	repair	Sult1d1
Cox7b	5.08E-06	0.301881	0.757	0.644	0.044022	repair	Cox7b
D8Ertd738e	5.77E-06	0.445008	0.454	0.318	0.050033	repair	D8Ertd738e
Vps29	7.85E-06	0.556694	0.294	0.181	0.068071	repair	Vps29
Atp6v1e1	9.89E-06	0.343692	0.456	0.318	0.085676	repair	Atp6v1e1
Ascc1	1.03E-05	0.392587	0.475	0.345	0.08936	repair	Ascc1
S100a1	1.15E-05	0.395252	0.54	0.405	0.09955	repair	S100a1
Glul	1.32E-05	0.539473	0.35	0.23	0.114715	repair	Glul
Gabarapl2	2.03E-05	0.437307	0.337	0.216	0.175793	repair	Gabarapl2
Mtch2	2.15E-05	0.513397	0.293	0.181	0.186254	repair	Mtch2
Gnas	2.93E-05	0.369635	0.42	0.293	0.253742	repair	Gnas
Dhrs4	3.08E-05	0.4421	0.446	0.334	0.267283	repair	Dhrs4
Dbp	3.64E-05	0.476696	0.315	0.2	0.31538	repair	Dbp
Park7	3.67E-05	0.299064	0.761	0.671	0.317764	repair	Park7
Gng5	4.03E-05	0.333846	0.671	0.564	0.349131	repair	Gng5
Map1lc3b	4.22E-05	0.442801	0.375	0.252	0.366047	repair	Map1lc3b
Uqcrh	4.30E-05	0.26085	0.77	0.69	0.37235	repair	Uqcrh
RP23-306P12-2	4.62E-05	0.417384	0.46	0.342	0.400547	repair	RP23-306P12-2
Adhfe1	5.79E-05	0.443363	0.321	0.208	0.501996	repair	Adhfe1
D630029K05Rik	5.97E-05	0.578669	0.263	0.164	0.517788	repair	D630029K05Rik
Gatm	6.30E-05	0.377972	0.542	0.419	0.546	repair	Gatm

Cat	6.37E-05	0.367029	0.547	0.427	0.551925	repair	Cat
Ndufab1	7.01E-05	0.303567	0.622	0.51	0.607631	repair	Ndufab1
Cryz	7.96E-05	0.418585	0.498	0.381	0.690243	repair	Cryz
Ech1	8.74E-05	0.419608	0.532	0.436	0.757109	repair	Ech1
Qdpr	8.83E-05	0.395495	0.476	0.351	0.765097	repair	Qdpr
Bri3	8.90E-05	0.380534	0.528	0.408	0.770991	repair	Bri3
Lamtor5	9.24E-05	0.398495	0.444	0.323	0.801113	repair	Lamtor5
Camk2n1	0.000104	0.497372	0.338	0.236	0.89859	repair	Camk2n1
Suclg1	0.000108	0.342491	0.525	0.408	0.932454	repair	Suclg1
Atp5f1	0.000138	0.354108	0.591	0.493	1	repair	Atp5f1
Slc25a4	0.000184	0.294555	0.403	0.288	1	repair	Slc25a4
Ndufb5	0.000188	0.319303	0.529	0.416	1	repair	Ndufb5
Hebp1	0.000219	0.497011	0.276	0.181	1	repair	Hebp1
Glud1	0.000223	0.436411	0.3	0.2	1	repair	Glud1
Hspd1	0.000229	0.321768	0.383	0.268	1	repair	Hspd1
Grhpr	0.000232	0.409451	0.473	0.364	1	repair	Grhpr
Gclm	0.000239	0.410516	0.427	0.321	1	repair	Gclm
Mrpl54	0.000302	0.518707	0.261	0.17	1	repair	Mrpl54
Eno1	0.000366	0.361866	0.466	0.351	1	repair	Eno1
Txn1	0.000374	0.264769	0.719	0.63	1	repair	Txn1
Stard10	0.000376	0.271087	0.42	0.312	1	repair	Stard10
Cmpk1	0.000399	0.347426	0.311	0.211	1	repair	Cmpk1

Polr3e	0.000425	0.529604	0.256	0.173	1	repair	Polr3e
Ppdpf	0.000431	0.54371	0.284	0.189	1	repair	Ppdpf
Dnajc12	0.000437	0.347536	0.503	0.405	1	repair	Dnajc12
Hsbp1	0.000457	0.341848	0.399	0.296	1	repair	Hsbp1
Mrps14	0.000458	0.465681	0.272	0.178	1	repair	Mrps14
Rnf181	0.00046	0.293498	0.47	0.364	1	repair	Rnf181
Timm13	0.000538	0.269814	0.564	0.447	1	repair	Timm13
Calm3	0.000538	0.38432	0.458	0.345	1	repair	Calm3
Gcsh	0.000559	0.436152	0.3	0.211	1	repair	Gcsh
Pdha1	0.000586	0.481053	0.315	0.225	1	repair	Pdha1
Cotl1	0.000631	0.435841	0.263	0.178	1	repair	Cotl1
Mpc2	0.00064	0.298243	0.687	0.616	1	repair	Mpc2
Gng12	0.000727	0.35999	0.297	0.2	1	repair	Gng12
Myeov2	0.000775	0.307305	0.54	0.444	1	repair	Myeov2
S100a10	0.000802	0.277689	0.264	0.175	1	repair	S100a10
Mrps21	0.00086	0.328376	0.59	0.501	1	repair	Mrps21
Coq9	0.000902	0.370461	0.274	0.186	1	repair	Coq9
Acads	0.000908	0.42103	0.322	0.23	1	repair	Acads
Mrpl50	0.000919	0.418711	0.259	0.175	1	repair	Mrpl50
Timm17a	0.000949	0.378908	0.277	0.184	1	repair	Timm17a
Snx3	0.001027	0.289262	0.311	0.216	1	repair	Snx3
Gipc2	0.001048	0.285337	0.283	0.195	1	repair	Gipc2

Fkbp1a	0.001192	0.286895	0.32	0.225	1	repair	Fkbp1a
Ndufs7	0.001363	0.290282	0.52	0.419	1	repair	Ndufs7
Grpel1	0.001411	0.458431	0.267	0.189	1	repair	Grpel1
Hdgf	0.00162	0.322237	0.283	0.195	1	repair	Hdgf
Tpd52	0.00214	0.35281	0.252	0.17	1	repair	Tpd52
Tomm5	0.002275	0.417959	0.293	0.214	1	repair	Tomm5
Pter	0.002352	0.309025	0.433	0.345	1	repair	Pter
S100a11	0.002379	0.266676	0.353	0.26	1	repair	S100a11
Serp1	0.002585	0.332233	0.27	0.192	1	repair	Serp1
Ddah1	0.002806	0.282444	0.367	0.277	1	repair	Ddah1
Etfb	0.002953	0.284153	0.68	0.611	1	repair	Etfb
Taldo1	0.003532	0.325016	0.314	0.236	1	repair	Taldo1
Hadh	0.003533	0.269535	0.508	0.419	1	repair	Hadh
Mrpl30	0.003837	0.316071	0.291	0.211	1	repair	Mrpl30
Ndufs3	0.003989	0.252901	0.364	0.271	1	repair	Ndufs3
Swi5	0.004011	0.289304	0.412	0.321	1	repair	Swi5
Dynlrb1	0.004343	0.388003	0.294	0.219	1	repair	Dynlrb1
Lypla1	0.004654	0.313095	0.419	0.345	1	repair	Lypla1
Pbld2	0.004838	0.338243	0.257	0.184	1	repair	Pbld2
Mcee	0.00562	0.347787	0.271	0.197	1	repair	Mcee
1110008F13Rik	0.005825	0.389334	0.271	0.203	1	repair	1110008F13Rik
Erich4	0.005977	0.36976	0.311	0.236	1	repair	Erich4

Dnaja1	0.007713	0.293895	0.327	0.252	1	repair	Dnaja1
Ankrd13c	0.007856	0.255113	0.315	0.241	1	repair	Ankrd13c
1810058I24Rik	0.008434	0.252599	0.407	0.321	1	repair	1810058I24Rik
Ndufa10	0.008487	0.324353	0.354	0.282	1	repair	Ndufa10
Aldh9a1	0.009453	0.262211	0.28	0.211	1	repair	Aldh9a1

Supplementary Table S2 The differentially expressed genes analysis results between repaired and atrophy PT cells of GSE161758.

PCR primers for qRT-PCR	
Genes	PCR primers
Cd74 F	5‘-AGTGCGACGAGAACGGTAAC -3’
Cd74 R	5‘-CGTTGGGGAACACACACCA -3’
mouse actin- F	5‘-AACAGTCCGCCTAGAAGCAC -3’
mouse actin -R	5‘-CGTTGACATCCGTAAAGACC -3’
App F	5‘-AGGACTGACCACTCGACCAG -3’
App R	5‘-CGGGGGTCTAGTTCTGCAT -3’

Supplementary Table S3 The primer sequences used in PCR of this research

Cell type	Macro	LOH-DL	LOH-AL	PT	CD-IC	DCT	Endo	CD-PC	Pod	Act-Fib1	Act-Fib2	URO	T cell
Marker genes													
	Ctss	Slc12a1	Slc12a1	SLC27A2	Slc26a4	Slc12a3	Emcn	Tmem45b	Nphs1	Pdgfrb	Pdgfrb	Upk1b	Cd247
	Itgam	Umod	Umod	Lrp2	Atp6v1g3		Ednrb	Aqp2	Nphs2	Dcn	Dcn		Cd3e
	C1qc	Epha7		Miox	Kit		Ehd3	Hsd11b2	Podxl	Cfh	Cfh		Cd8a
	C1qb	Bst1		Slc4a4	Slc4a1		Igfbp3	Fxyd4		Tnc	Tnc		Trbc2
	C1QA				Aqp6		Plat	Aqp3		Spon1	Spon1		Thy1
	Lyz2						Tek	TRPV5		Bmpr1b	Bmpr1b		
										Vcan			

Supplementary Table S4 The cell markers used in the cell type identification.
