# Supplementary material for: Cell Profiling of Acute Kidney Injury to Chronic Kidney Disease Reveals Novel Oxidative Stress Characteristics in the Failed Repair of Proximal Tubule Cells
Source: Int J Mol Sci. 2023 Jul 18;24(14):11617. doi: 10.3390/ijms241411617 (PMC10380716; doi:10.3390/ijms241411617)
Supplement: Supplementary file 1 [file ijms-24-11617-s001.zip › ijms-2393050-supplementary.pdf]

Supplementary details about Module score calculation

We first analyzed correlations between inferred module score and target cell score by identifying a set of genes for each cell type using the average expression of genes for that cell type in neonatal kidney ScRNA data. Genes were considered specifically expressed if they exhibited an expression value greater than 0.8 (Seurat normalized values) in the cell type of interest and less than 0.2 in all other cell types. These gene sets were then used to calculate a cell type module score, which was computed by finding a set of top 200 background genes in the same expression bin for each gene in the signature and subtracting the mean expression of this background gene set from the mean expression of the cell type gene set in our SnRNA data.
